# History of Shrimp Farming and the Main Viral and Bacterial Diseases in Mexico

**DOI:** 10.3390/microorganisms13112631

**Published:** 2025-11-20

**Authors:** Cesar Marcial Escobedo-Bonilla, Jareli Itzel Colula-Ocampo, Rosa Idalia Hernández-Herrera, Martina Hilda Gracia-Valenzuela, Pablo San Martín del Ángel

**Affiliations:** 1 Instituto Politécnico Nacional-CIIDIR Unidad Sinaloa, Blvd., Juan de Dios Batiz Paredes No. 250, Colonia San Joachin, Guasave 81101, Sinaloa, Mexico; 2 Facultad de Ciencias Biológicas y Agropecuarias, Universidad Veracruzana, Carr. Tuxpan-Tampico km 7.5, Col. Universitaria, Tuxpan 92860, Veracruz, Mexico; jareliclm@gmail.com (J.I.C.-O.); idhernandez@uv.mx (R.I.H.-H.); pmartin@uv.mx (P.S.M.d.Á.); 3 Tecnológico Nacional de México-Instituto Tecnológico del Valle del Yaqui, Ave. Tecnológico, Block 611, Valle del Yaqui, Bácum 85276, Sonora, Mexico; mgracia.valenzuela@itvy.edu.mx

**Keywords:** aquaculture, shrimp farming, virus diseases, bacterial diseases, history, Mexico

## Abstract

Shrimp farming is probably the most recent animal production activity generating high-quality animal protein, jobs, and economic revenue for many developing and middle-income countries worldwide. Despite the steady growth over the last two decades, aquaculture production has recently seen a decreased growth rate, with infectious diseases being a culprit. Mexico is a major farmed shrimp producer in the world, with the industry generating about USD 1 billion each year and providing jobs for those in vulnerable social sectors. Nonetheless, various viral and bacterial diseases have affected production and hampered development. This review provides a historic account of shrimp farming in Mexico and the chronology, features, and impact of the main infectious diseases. At present, *Penstylhamaparvovirus* (formerly known as IHHNV) has decreased in importance as a pathogen and is the only virus that has coexisted with other major pathogens. In Mexico, main pathogens dominate shrimp farming during certain periods, then they become eclipsed by a new emergent pathogen. Exotic pathogens likely entered Mexico through the movement of live broodstock or larvae or through contaminated imported crustacean commodities for human consumption. Stronger biosecurity measures such as quarantine protocols and sensitive diagnostic tools at the borders are required to reduce the risk of other emergent pathogens.

## 1. Importance of Aquaculture

Aquaculture has become an important industry in aquatic animal products for human consumption, since global demand for protein of aquatic animal origin has increased faster than supplies from capture fisheries. This has made aquaculture a leading source of aquatic animal protein worldwide. In 2022, aquaculture production of aquatic animals surpassed capture fisheries for the first time [1].

Aquaculture and fisheries employ 61.8 million people globally. Of these, 36% (22.24 million people) are in aquaculture, and women hold 62% of processing jobs [1]. In Mexico, the shrimp farming production chain (hatcheries, farms, processing, logistics) supports around 20,000 direct (farm pond) and indirect (processing and trading) jobs, generating over USD 1 billion annually, with the U.S. absorbing around 80% of the export volume [2].

Although Asian countries generate over 90% of farmed aquatic animals, Latin America has emerged as the second-largest aquaculture region with 3% of global production with crustaceans as its main driver [1]. Latin America has built an export-orientated aquaculture industry due to its geographical conditions, a climate allowing year-round culture cycles, and its proximity to the U.S. and European markets. Shrimp aquaculture is one major industry which has rapidly expanded [3]. In Latin America, Ecuador, Mexico, and Brazil are the main suppliers of farmed shrimp in the region. Mexico is the second-largest farmed shrimp producer in Latin America and the seventh worldwide (Table 1) [4].

Shrimp is a valuable commodity with high nutritional value. It contains a significant amount of high-quality protein (24%), carbohydrates (0.2%), fats (0.28%), calories (0.99 per g), and other micronutrients [5]. Worldwide, aquatic foods provide more than 20% of animal protein intake for 3.2 billion people [1]. In Mexico, shrimp consumption per capita (~2 kg per year) is double the average for other crustaceans [6].

The methodology to produce this review was to search in online public (google, latindex, dialnet, Scielo) and private (Elsevier, Wiley, Springer) databases to find over 200 published papers, as well as book chapters and technical manuals, dealing with the presence of viral and bacterial diseases worldwide and in Mexico. The sources were reports from official government bodies, international organizations directly involved with shrimp aquaculture, and Mexican and international researchers who had worked with diseases in Mexico.

## 2. Global Farmed Shrimp Production

Global aquaculture production peaked in the 1980s and 1990s at annual growth rates as high as 11 and 10%, respectively. In the decade 2006–2016, aquaculture production saw reduced growth at rates of 5.8% but, nonetheless, its growth remained higher than other major animal production sectors [7]. In 2016, world fish production for human consumption was 177 million tons worth USD 362 billion. Of this production, 47% (~80 million tons) came from aquaculture [7]. In 2022, global fish production increased to 223.2 million tons, of which 185.4 million tons were aquatic animals and 37.8 million tons were algae. Most of the aquatic animal production (89%) was for human consumption [1]. That year, animal aquaculture production (94.4 million tons) exceeded (51%) fishery production for the first time [1].

Crustacean aquaculture has rapidly developed in recent decades, becoming the third most farmed group, with shrimp being the single most cultured crustacean by volume (estimated in 8.3 million tons), reaching 59.6% of the global crustacean production in 2023 [4]. In 2022, farmed crustaceans accounted for 13.5% of total aquaculture global production, increasing by 1.6 million tons compared to 2020 [1]. Crustacean aquaculture is an important industry mainly dominated by Asian countries, and Latin America is a key contributor of farmed shrimp.

Penaeid shrimp aquaculture is a major animal production industry in several developing countries in Asia, Latin America, Africa, and the Middle East [1,8]. The main farmed shrimp species have changed according to certain biological and culturing features [9,10]. Until 2004, the main farmed species was the Asian black tiger shrimp *Penaeus monodon*. Since then, the American whiteleg shrimp *Penaeus vannamei* has become the most cultured species worldwide due to its introduction and rearing in Asian countries such as China, India, and Thailand. In 2010, *P. vannamei* accounted for 71.9% of the world farmed shrimp production. In 2023, this species accounted for 89.5% of the total volume, while the giant black tiger shrimp accounted for 9.9% [4].

Some traits that make *P. vannamei* the preferred species for aquaculture both in America and Asia are its remarkable ability to acclimatize to broad environmental variations (salinity, temperature) and its good performance under culture conditions (growth rate, high stocking densities) and even in conditions of limited food diversity. This relates to its omnivorous feeding habits that include organic matter and detritus from plants and animals. Moreover, *P. vannamei* has a relatively low dietary protein requirement (25% vs. 40% in *P. monodon*) and good feed conversion rate efficiency. These features make it an attractive species for aquaculture [9,10,11].

In 2023, the main shrimp farming countries (*n* = 13) were Asian (*n* = 8) and Latin American (*n* = 5) (Table 1). In 2023, the Pacific white shrimp *P. vannamei* accounted for 7.2 million tons, while the Black Tiger prawn *P. monodon* reached 799,766 tons and the Kuruma prawn *P. japonicus* produced 47,304 tons. Other species such as the Banana prawns (*P. merguiensis* and *P. indicus*) and the blue shrimp *P. stylirostris* had marginal production [4].

## 3. Shrimp Farming in Latin America

Shrimp aquaculture started in 1934 in Japan with the technology developed by Dr Fuginaga to produce postlarvae from eggs spawned from wild brooders of *P. japonicus* [12,13,14]. He also developed the grow-out technology for shrimp aquaculture in the early 1960s [13,14,15,16].

The breeding place for Latin American shrimp farming was the U.S. in the 1960s, as Fuginaga’s methods were used to develop rearing techniques for American shrimp species. The Galveston Laboratory developed a clear water method to rear Gulf of Mexico shrimp species (*P. aztecus*, *P. duorarum*, and *P. setiferus*), whereas in Florida, hatchery and grow-out methods were developed to culture the Atlantic pink shrimp *P. duorarum* [13,14].

In 1967, a shrimp hatchery was established in Florida which had a monthly production of between 50 and 100 million postlarvae of the species *P. setiferus*, *P. aztecus*, and *P. duorarum*, having the best results with the former species [13,14]. Between 1968 and 1972, that hatchery produced *P. duorarum* postlarvae from wild spawners and stocked them in coral canals and small ponds in the Florida Keys. Better survival was reported in ponds compared to canals, but in both cases the species showed low growth. Later, nauplii of *P. stylirostris* and *P. vannamei* were imported from Nicaragua and Panama, obtaining good results. Grow-out was performed initially in an extensive culture system using a 1000 ha bay and later two 120 ha ponds. Although this project was not profitable, it produced up to 375 tons per year [13]. In 1970, a shrimp farm research center in Florida compared culture traits of various American species: *P. duorarum*, *P. aztecus*, *P. setiferus*, *P. schmitti*, *P. brasiliensis*, *P. occidentalis*, *P. stylirostris*, *P. vannamei*, *P. californiensis*, and *P. paulensis*. The blue shrimp *P. stylirostris* showed better culture performance due to a faster growth rate, larger harvest size, docility, and easier maturation [11,13].

In the late 1960s and early 1970s, shrimp farming started out in various countries in Latin America [15]. In the 1960s, commercial shrimp grow-out farms started in Ecuador, using as the main species *P. vannamei* and *P. stylirostris*. The industry expanded thanks to the abundance of wild postlarvae [14]. In the early 1970s, shrimp aquaculture was extensive, using large (20–100 ha) ponds stocking wild *P. vannamei* at low density and with no supplemental feed. This system was profitable since little economic input was required and labor was cheap. By 1977, around 3000 ha. were dedicated to extensive shrimp farming in Ecuador [13]. In the mid-1970s, water-stable shrimp feeding trials were performed in Ecuador. The introduction of artificial feed greatly improved growth, survival, and production. This prompted the development of shrimp feed mills in the 1980s and set the scene for the evolution of Ecuadorian shrimp farming from extensive to semi-intensive production. In 1983, shrimp production increased to 23,390 tons from 4800 tons in 1978. Nonetheless, the industry was still dependent on wild postlarvae. Weather phenomena like “La Niña” caused the decline of wild shrimp larvae [13]. This promoted the artificial production of shrimp postlarvae through hatcheries to reduce the dependence on wild larvae. Hatcheries prompted the growth of the Ecuadorian shrimp industry [13,14].

The semi-intensive culture system from Ecuador became the development model for shrimp farming in various countries in the Americas such as Panama, Costa Rica, Honduras, Colombia, Mexico, and Brazil using as farming species *P. vannamei*, *P. schmitti*, and *P. brasiliensis*. In 1968, a shrimp farm was developed on the Atlantic coast of Honduras which produced *P. aztecus*, *P. stylirostris*, and *P. occidentalis* from Panama. The latter species showed low growth and survival. In 1974, a hatchery and a semi-intensive shrimp farm were established in Panama, successfully rearing *P. stylirostris* [11,13]. From 1984 to 1992, a leading farm increased its culture area to a total of 3600 ha. In 1984, Panama had various shrimp farms of more than 2000 Ha and mainly farmed *P. vannamei* and *P. stylirostris* due to their fast growth and good survival [17]. In 1978, farmers in Panama preferred *P. vannamei* over *P. stylirostris* due to better maturation, spawning conditions, and nutritional requirements [11]. In the mid-1970s, the first shrimp farm was built in Costa Rica. In Brazil, a leading farm imported and domesticated *P. vannamei* and *P. stylirostris* from Panama and *P. monodon* and *P. penicillatus* from a laboratory in Taiwan [13].

## 4. Shrimp Farming in Mexico

The origins of shrimp aquaculture in Mexico date from the late 1960s and early 1970s, when an agreement was signed between the Center for Scientific and Technological Research at the University of Sonora (CICTUS, today DICTUS) and the University of Arizona [18,19]. In 1969, the first culture of Pacific brown shrimp (*P. californiensis*) was performed under controlled conditions at the facilities of a private university at Guaymas Sonora [20,21,22]. In the 1970s the first culture experiments were performed at CICTUS with blue shrimp (*P. stylirostris*), brown shrimp (*P. californiensis*), and Pacific white shrimp (*P. vannamei*) using intensive methods [23]. After good results were obtained with the blue and white shrimp, CICTUS began experiments at its Kino Experiment Station, using extensive and semi-intensive culture systems. Production rates of 0.77–0.81 kg m^−2^ were obtained in 200 m^2^ earthen ponds [18,19,24,25]. By 1975, culture experiments with *P. stylirostris* were being performed using semi-intensive and extensive conditions [20,24]. In 1977, a 7.5 ha. experimental semi-intensive farm for the culture of *P. stylirostris* was constructed at Puerto Peñasco Sonora, as part of the University of Sonora [26].

In the late 1970s and early 1980s, only a few cooperatives and some private producers developed shrimp farms in Sonora and Sinaloa. At this time, the exploitation of seafood species was reserved for the social sector (cooperativas or ejidos). This meant that the private sector had little opportunity to invest in these areas [19,22,27]. Major changes to fishery laws occurred in 1986 when requirements for the formation of cooperatives were eased and a mechanism by which private sector could invest in fisheries was installed in agreement with the cooperatives. These changes did not increase private investment in aquaculture until another substantial change occurred in 1992. Here, article 27 of the Mexican Constitution dealing with land tenure and the Fisheries Laws were changed. This allowed peasants to own the land and sell it, so private investors could buy land and build aquaculture facilities [19]. These actions prompted the development of commercial shrimp farming [19,22].

**Figure 1:**
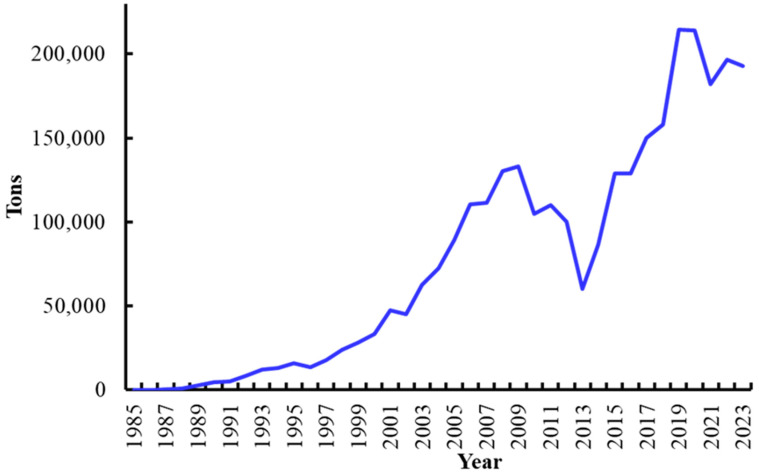
Farmed shrimp production (weight) in Mexico in the period 1985–2023 [6,31].

Commercial shrimp farming in Mexico started in 1985 [19,28,29] when 42 shrimp farms were established in northwest Mexico out of a total of 49 nationwide [30]. That year farmed shrimp production was 35 tons [31] and gradually increased both in number of farms and volume produced (Figure 1) [4,6,31]. Shrimp farming is mainly carried out in northwest Mexico (Sonora, Sinaloa, Nayarit, and Baja California Sur), where about 95% of the shrimp farming surface is located and where 95% of the total shrimp farming volume is produced [6,22,26,29,32].

At the beginning, commercial shrimp farming in northwest Mexico mostly used the extensive culture system [20,26,27,33]. Extensive cultures were often performed in natural water bodies such as estuaries, just enclosing the arriving postlarvae and providing no feed [33]. The extensive system was also used in large ponds which varied between 8 to over 100 ha, and the stocking density was <10 shrimp m^−2^ [19,24,27,32]. Stocking occurred when wild postlarvae arrived at coastal estuaries and were caught by fishers between February and April (spring season). Postlarvae varied in age and size and could belong to the species *P. vannamei*, *P. stylirostris*, and *P. californiensis*, with the former species often being the most abundant [17].

In the late 1980s and early 1990s, many shrimp farmers switched to the semi-intensive culture system [19,20,32]. The semi-intensive system is employed in ponds between 2 and 10 ha, with stocking densities between 20 and 50 shrimp m^−2^. In the early 1990s semi-intensive systems were a small fraction of total shrimp farming operations. In 1996, Mexico had 284 shrimp farms, of which 77 (27%) used the extensive system with 18% of the culture surface, 196 (69%) employed the semi-intensive system with 78% of the culture surface, and the remainder 11 (4%) used the intensive system [19,20,26,32,34]. Since 2008, the semi-intensive culture system has dominated (~85%) in the country. That year in Sonora, 96% of shrimp farms used the semi-intensive culture system, and 4% used the intensive one. In Sinaloa 89% of shrimp farms utilize the semi-intensive system, 9% use the extensive system, and 2% employ the intensive system. In Nayarit 70% of shrimp farms use the semi-intensive system, 28% employ the extensive system, and 2% use the intensive system [29,35].

The intensification of shrimp farming has increased the number of stress factors, resulting in enhanced susceptibility to diseases [36,37,38]. Environmental stressors include variations in temperature or salinity due to heavy rain. Also, toxic substances in water from industrial or agricultural sources have been associated with the development of disease and mortality [39,40]. Intrinsic farming factors such as pond overcrowding, overfeeding, nutritional requirements not being met, and poor water quality have been involved in disease onset [41].

Infectious diseases have been associated with shrimp farming since its beginnings [42,43] and are possibly the main threat hampering its development [8,44,45,46]. Infectious diseases are a major issue affecting farmed shrimp production worldwide.

The presence of infectious viruses in crustaceans was first reported in 1966 in the mediterranean crab *Portunus depurator* [47]. Afterwards, other viruses were reported infecting decapod crustaceans. The main pathogens causing infectious diseases in farmed shrimp are viruses, which are believed to cause 60% of global production losses; bacterial diseases have been blamed for an additional 20% losses, and the remaining 20% of losses have been attributed to fungal infections [38]. In Mexico, shrimp farming has experienced changes both in cultured species and stocking densities as a result of the appearance of infectious diseases mainly of viral and bacterial etiology.

## 5. Major Shrimp Diseases in Mexico

### 5.1. Baculovirus penaei (Penaeus vannamei Singly Enveloped Nuclear Polyhedrosis Virus)

*Baculovirus penaei* (BP), also called *Penaeus vannamei* Singly Enveloped Nuclear Polyhedrosis Virus (PvSNPV) [48,49,50], was the first reported virus infecting the penaeid shrimp *P. duorarum* from the Gulf of Mexico in 1974 [51]. It was later found that PvSNPV infects at least three Asian and nine American shrimp species including *P. stylirostris* and *P. vannamei* [50,52,53,54]. Before 1987, reports of PvSNPV in wild and farmed penaeid shrimp occurred in the U.S. from Florida to Texas in the Northern Gulf of Mexico. In the Americas, the virus was described in Brazil, Peru, Ecuador, Panama, and Costa Rica [55]. Recently PvSNPV was found in farmed *P. vannamei* in Taiwan [56]. Other host species include *P. aztecus*, *P. marginatus* [53], *P. schmitti*, *P. penicillatus*, and *P. subtilis* in Brazil [57].

This pathogen is a rod-shaped enveloped virus with a genome of double-stranded DNA [53]. The virion is 312–320 nm long and 75–87 nm wide. The polyhedrin subunits are 17–19 nm in diameter [49]. Although PvSNPV was described over 50 years ago, its full genome sequence was just recently elucidated. Its genome is a circular, double-stranded DNA molecule with 119,883 bp encoding 101 Open Reading Frames (ORFs). Phylogenetic analyses of amino acid sequences clustered PvSNPV with *Penaeus monodon* nudivirus (MBV) [58].

**Figure 2:**
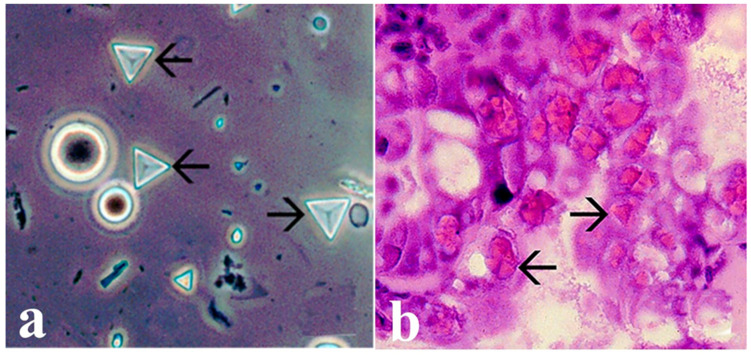
(**a**) Tetrahedral occlusion bodies (arrows) of a feces wet mount from Pacific white shrimp (*P. vannamei*) infected with PvSNP. (**b**) Pacific white shrimp postlarva with grade (3–4) PvSNPV infection. Eosinophilic tetrahedral baculovirus OBs within hypertrophied nuclei in cells of hepatopancreas (arrows). Photos from AGDAFF–NACA (2007) [60]. Magnification 700×.

The virus replicates in epithelial cells of the hepatopancreas and midgut in susceptible penaeid species [51,52,53]. Cellular changes in PvSNPV infection include reduced chromatin and its margination, nuclear hypertrophy, nucleolar degeneration, and the formation of intranuclear tetrahedral occlusion bodies (OBs) (Figure 2) enclosing hundreds of rod-shaped enveloped virions. The OBs are polyhedrin structures enclosing and protecting enveloped infectious virions. The presence of these OBs represents evidence of PvSNPV infection [50,52,53].

The oral route is the transmission pathway for the virus, since OBs and free virions are released into the lumen of the digestive tract as result of the necrosis and lysis of infected epithelial cells of the hepatopancreas or midgut and they exit to the water with feces, thus increasing the risk of horizontal transmission by shrimp eating virus-infected materials or tissues [52,53,54]. The size of OBs in *P. stylirostris* and *P. vannamei* ranges from 8 to 10 µm [53].

Clinical signs of infection include reduced feeding, preening, and activity, decreased growth, and increased epibiont fouling [53,59]. PvSNPV affects all life stages of affected penaeid shrimp, from protozoa III until the adult stage [36,53], but in *P. vannamei* and *P. stylirostris*, the virus is often associated with the postlarval and early juvenile stages in hatcheries and grow-out ponds [36,50,53]. In wild *P. duorarum* and *P. aztecus*, the most affected stages are juvenile to adult [51,53].

In shrimp larval culture, PvSNPV has been the only virus confirmed to cause mortality (Protozoea to postlarva stages) [36]. In Mexico, the first report of PvSNPV occurred in 1987 in a hatchery of blue shrimp *P. stylirostris* in northwest Mexico [55]. In the early 1990s, PvSNPV was a problem in hatcheries, where the virus affected the protozoea III to postlarva larval stages [53]. Nonetheless, the incidence of PvSNPV in hatcheries has significantly reduced due to nauplii and zoea from suspected PvSPNV-infected broodstock being disinfected. Also, the use of virus-free brooders has reduced the risk of PvSNPV outbreaks in hatcheries and grow-out ponds [36]. At present, PvSNPV seldom occurs in hatcheries and grow-out ponds as result of the preventive measures adopted.

### 5.2. Infectious Hypodermal and Hematopoietic Necrosis Virus (IHHNV) (Penstylhamaparvovirus 1)

This virus was named Infectious Hypodermal and Hematopoietic Necrosis Virus (IHHNV) to describe the damage caused in *P. stylisrostris* [61]. Later, due to its genomic organization and resemblance to the parvovirus subfamily, it was called *Penaeus stylirostris* densovirus (PstDNV) [62] and recently renamed as Decapod *Penstylhamaparvovirus* 1 and moved to the new subfamily Hamaparvovirinae within the family *Parvoviridae* [63].

The origin of *Penstylhamaparvovirus* 1 (IHHNV) seems to be from Asian shrimp species (*P. monodon* and/or *P. japonicus*) [64]. Later, molecular studies in various *Penstylhamaparvovirus* isolates from Asia and the Americas showed that a Philippine IHHNV isolate clustered with IHHNV found in the Western Hemisphere [65], thus strengthening the hypothesis of its Asian origin. Introductions of IHHNV-infected *P. monodon* stocks from the Philippines into the Americas occurred during the mid-1970s to early 1980s [13,64,65]. A commercial semi-intensive farm and hatchery in Panama where mainly *P. stylirostris* was cultured may have been the initial site of *Penstylhamaparvovirus* entry into the Americas. The virus caused high mortality to *P. stylirostris* but not to *P. vannamei* [13,64,66].

In 1981, the impact of *Penstylhamaparvovirus* was first reported in Hawaii from batches of *P. stylirostris* that had come from Central and South America under semi-intensive and intensive cultures [65,66,67] and also in stocks of *P. vannamei* [64,68]. This virus rapidly spread to other countries in the Americas (Mexico, Panama, Colombia, Ecuador, and Argentina) [69,70,71,72], Asia (Indonesia, Malaysia, Singapore and Thailand) [69,73], and French Polynesia [74].

*Penstylhamaparvovirus* 1 is the smallest virus infecting shrimp (22 nm average diameter). The virion has an icosahedral shape and is non-enveloped, with a density of 1.4 g/mL in CsCl [74]. Its genome is a single-stranded DNA with a size between 4100 and 4700 bases [75]. The genome is organized into three open reading frames (ORFs) encoding a non-structural protein, an unknown protein, and a capsid protein, respectively [76]. The replication of *Penstylhamaparvovirus* 1 is slower than that of WSSV. Upon intramuscular inoculation, *Penstylhamaparvovirus*-infected *P. vannamei* become positive by PCR after 7 days [77].

Natural *Penstylhamaparvovirus* infection has been reported in several shrimp species: *P. stylirostris*, *P. vannamei*, *P. occidentalis*, *P. californiensis*, *P. chinensis*, *P. monodon*, *P. semisulcatus*, *P. japonicus*, and *Artemesia longinaris* [64,72,73]. Experimental infections have also been described for *P. setiferus*, *P. aztecus*, and *P. duorarum*. In contrast, *P. indicus* and *P. merguiensis* appear to be refractory to *Penstylhamaparvovirus* infection [59,64].

This virus is transmitted horizontally by exposure to contaminated water, through *per os* ingestion of infected shrimp, and by direct inoculation of the virus [53]. The virus is probably shed with eggs from infected broodstock, and the virus may linger around larval stages until susceptible postlarvae become infected [61,78].

Histopathology of *Penstylhamaparvovirus* infection shows cells with nuclear hypertrophy as result of formation of eosinophilic Cowdry-Type A intranuclear inclusions and marginated chromatin. These intranuclear inclusion bodies may be confused with the early-stage intranuclear inclusion bodies observed in WSSV infection. Infected cells undergo pyknosis and karyorrhexis, leading to areas of multifocal necrosis in organs of ectodermal (nervous tissues, fore- and hindgut, integument, gills, antennal gland), and mesodermal (hematopoietic tissue, striated muscle, heart, gonads, lymphoid organ, connective tissues) origin [53,79,80].

**Figure 3:**
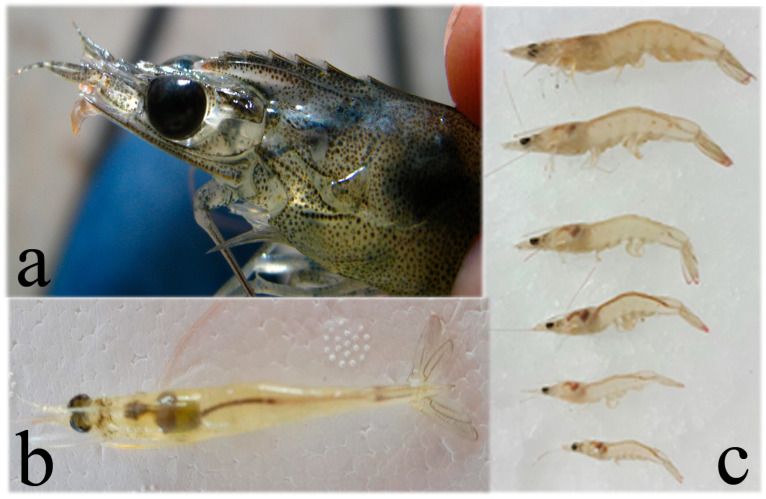
Runt deformity syndrome (RDS) shown as (**a**) deformed rostrum and (**b**) body deformity in *P. vannamei* acutely infected with *Penstylhamaparvovirus*. (**c**) Clear size variation in *P. vannamei* infected with *Penstylhamaparvovirus*. All images from own sources.

Clinical signs of IHHNV infection depend on the species age and size; the early juvenile stages are more susceptible to the disease [69]. In *P. stylisrostris*, shrimp with acute IHHNV infection display reduced feeding and locomotion and behavioral changes during swimming, and at the end, they sink to the pond bottom and die due to the infection [53,61,69]. In *P. vannamei*, with acute *Penstylhamaparvovirus* infection show reduced growth rates and marked size differences within a pond population. Another outstanding feature is that infection causes deformity of the rostrum, antennae, and/or cuticle (Figure 3). These deformities are known as “runt deformity syndrome” (RDS) [81]. In other species such as *P. monodon*, *Penstylhamaparvovirus* infection apparently causes no harm at the clinical level as no differences in size, weight, or fertility have been found between IHHNV-positive animals (determined by PCR and/or *in situ* hybridization) compared to healthy ones. No inclusion bodies were observed in *Penstylhamaparvovirus*-positive farmed *P. monodon* [82,83].

In Mexico, the first *Penstylhamaparvovirus*-positive sample was detected in late 1987 from an imported batch of *P. vannamei* postlarvae from the U.S. delivered in Southern Sinaloa and Baja California Sur. Later, other farms located in the central and northern parts of the Gulf of California and imported postlarvae of *P. vannamei* and *P. stylirostris* from the U.S., Panama, and Ecuador were *Penstylhamaparvovirus*-positive. In 1989 and 1990, the virus caused huge mortality and economic losses in shrimp farms rearing *P. stylirostris* in Sonora and Sinaloa. At this time, the first wild stocks of postlarvae or juveniles *P. stylirostris* were found to be virus-positive [66,84]. Since 1987, this virus rapidly spread into the wild shrimp populations in the Gulf of California. This may be prompted by hatchery management practices of releasing excess (virus-infected) postlarvae into the wild to replenish wild shrimp stocks [66]. Then, virus-infected wild spawners are used to produce postlarvae to stock shrimp farms [79].

In Mexico, the *Penstylhamaparvovirus* outbreak in 1987–1990 caused severe damage to fisheries and aquaculture for blue shrimp *P. stylirostris*, which was then the main farmed species. This epizootic forced shrimp farmers to swap the highly IHHNV-susceptible species *P. stylirostris* for the less susceptible species *P. vannamei* [77,85].

In wild populations of *P. stylirostris*, the estimated prevalence of *Penstylhamaparvovirus* in adults varies between 80 and 89% in females and 57 and 60% in males by dot blot and histopathology [70]. Another study estimated the prevalence of *Penstylhamaparvovirus* in wild *P. stylirostris* of the Gulf of California by histopathology and *in situ* hybridization. The prevalence was 46% in the north and 26% in the central-southern region [86]. It was estimated that the economic impact of this virus was between USD 0.5 and 1.0 billion [68]. *Penstylhamaparvovirus* has become endemic in wild shrimp populations and shrimp farming facilities in northwest Mexico [79].

### 5.3. Taura Syndrome Virus (TSV) (Aparavirus Dicistroviridae)

This virus first appeared in shrimp farms near Taura river, Ecuador, in 1992. This location gave the virus its name. At first, clinical signs were thought to be caused by chemicals used against banana pests in nearby plantations, but in 1994, the viral etiology was confirmed [87]. Soon after its appearance, TSV spread to several countries in south, central, and north America, as well as to Hawaii [88]. Since 1999, TSV has also been detected in Asian countries such as Taiwan, Thailand, and Korea, which import stocks of *P. vannamei* from south America [89,90,91,92].

It is likely that TSV entered northwest Mexico through imported live broodstock and postlarvae from central and south America [19]. In early 1995, TSV was detected in wild adult *P. vannamei* collected off the Chiapas coast (southern Mexico), near the border with Guatemala, where TSV first appeared in Guatemalan shrimp farms in 1994. Soon after, TSV appeared in imported *P. vannamei* broodstock and postlarvae in at least two farms in northwest Mexico [79,93]. By September 1995, the TSV epizootic had spread to *P. vannamei* shrimp farms in Guerrero (southwest Mexico) and Sonora and Sinaloa (northwest Mexico) [93].

The TSV outbreak in Mexico has been one of the worst epizootics in Mexican shrimp farming, with estimated production losses between 37 and 80% [27,93,94]. In 1996, TSV prevalence varied from 67 to 95% in farmed and wild *P. vannamei* postlarvae and juveniles in Sinaloa [94].

In 1995–1996, *P. vannamei* was the main farmed shrimp species in Mexico (72%), whereas *P. stylirostris* was marginally cultured (7%). Due to TSV, the proportion of farmed blue shrimp increased to 25% [95]. Along with increasing the proportion of farmed blue shrimp, farmers stocked Specific Pathogen-Resistant (SPR) lines of blue shrimp [93]. This shrimp line (Super shrimp) developed in French Polynesia derived from blue shrimp surviving IHHNV epizootics. It was introduced in northwest Mexico as a strategy to reduce the impact of TSV as the species is naturally resistant to the virus [79].

Taura syndrome virus caused economic losses worth USD 2 billion between 1992 and 1996 throughout Latin America [11,96]. In Mexico, shrimp production losses caused by TSV between 1996 and 1998 were estimated at USD 35 million [97]. Although switching shrimp species and stocking SPR lines of blue shrimp or postlarvae from white shrimp surviving TSV were management strategies against the TSV epizootics [94,98,99,100], these shrimp lines suffered significant mortality due to white spot syndrome virus (WSSV) epizootics. In early 2000s, TSV was replaced by WSSV as the main pathogen in Mexican shrimp farms, leading TSV to virtually disappear.

TSV was isolated in 1997, and its biophysical and genomic properties were analyzed. The virion has an icosahedral shape, is non-enveloped, is 31–32 nm in size, and has a density of 1.34 g/mL in CsCl [101]. Its genome is a single, positive-sense RNA strand 10,205 nucleotides long [102]. The genome consists of two ORFs. The first one is 6740 nucleotides long and encodes a putative non-structural polyprotein with several domains such as a helicase, a protease, and an RNA-dependent RNA polymerase. The second ORF encodes three structural proteins, VP2, VP1, and VP3, in 3036 bases from nucleotides 6947 to 9982. These ORFs are separated by a 210-base non-coding intergenic region [102]. These biophysical and genomic features initially placed TSV as a member of the family *Picornaviridae* [103]. Later, the structure of its genome, the presence of a 230-nucleotide intergenic region with a similar non-AUG-mediated translation, containing an internal ribosome entry site (IRES), which directs the synthesis of the TSV capsid proteins, and a nucleotide sequence similar to the genus of *Cricket paralysis*-like viruses led TSV to be recognized as a member of the new family *Dicisctroviridae*, which includes small RNA viruses infecting insects [102,104]. The presence of the IRES element led TSV to be recently assigned to the new genus *Aparavirus*, within the family *Dicistroviridae*, which includes four viruses infecting bees and ants and two viruses infecting marine crustaceans [105].

**Figure 4:**
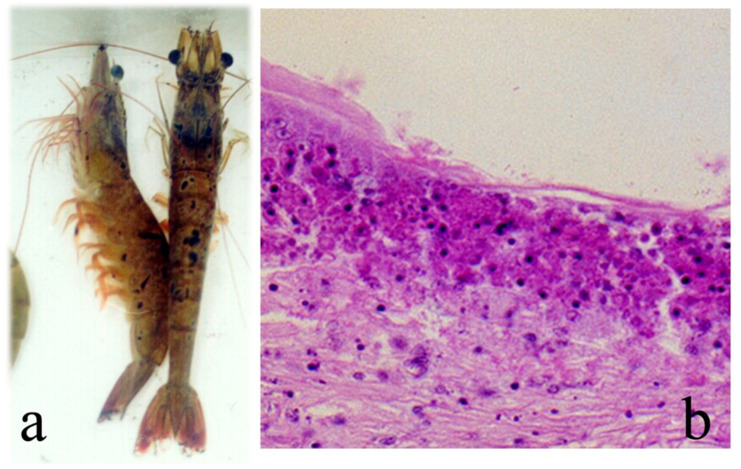
(**a**) Shrimp *P. vannamei* with acute TSV infection showing reddish discoloration of the body, pleopods, uropods, and telson, with multifocal necrosis in the cuticle (melanized spots). (**b**) Histopathological section (450×) showing TSV infected cells displaying pyknotic nuclei (dark spots) giving a “buckshot” appearance. Also, the presence of basophilic cytoplasmic TSV inclusion bodies and areas of necrosis in cuticular epithelium and subcuticular tissue in juvenile white shrimp with acute TSV infection. Histology photo from AGDAFF–NACA (2007) [60].

Clinical signs of TSV infection in juvenile *P. vannamei* include a soft loose exoskeleton, with expanded red chromatophores in appendages, uropods, and the telson (due to which TSV was also called red tail disease) but also on the body surface, appearing as a pale-orange discoloration [106], with multifocal necrosis of the cuticular epithelium (Figure 4), lethargy, abnormal swimming, a lack of appetite, and an empty gut [88,103].

Three clinical stages have been recognized in TSV infection: acute, transition, and chronic [96]. Shrimp in the acute stage display a soft exoskeleton, often with melanized multifocal necrosis and expanded chromatophores in the body, especially uropods and pleopods. Animals become weak and display an empty digestive tract. The acute stage is often related to the late premolt or early postmolt stages. Infected animals often die during molting, and cumulative mortality reaches 75–95% [69,96]. In the acute stage, cellular damages include pyknosis, karyorrhexis, and necrosis of epithelia in the cuticle, digestive tract, gills, antennal gland, and hematopoietic tissues. Infected cells appear as eosinophilic to pale basophilic spherical bodies, giving a peppered or buckshot appearance (Figure 4) [43,87,106]. The acute stage occurs from 3 to 5 days after the onset of infection. The transition stage occurs from 4 to 8 days after the onset of infection and is characterized by a reduction in the severity and number of infected cells. Hemocyte infiltration and the onset of melanization are observed. These features are indicative of a resolving acute phase [96]. Shrimp surviving the acute stage evolve towards a chronic stage which histologically shows wound repair (inflammation and fibrosis), leading to the regeneration of epithelial tissues in affected organs. By 8 days after infection, mortality ceases and surviving shrimp molt, shedding the necrotized cuticle. The lymphoid organ begins forming spheroids, or it shows a near-normal appearance [43,96]. Postlarvae and early juveniles of *P. vannamei* are the most susceptible stages to TSV, showing high mortality [87,106]. Size and age are factors for increased susceptibility to TSV infection in specific pathogen-free (SPF) *P. vannamei*, since larger animals are more susceptible to infection and mortality than early juveniles [107].

Shrimp species susceptible to TSV infection include *P. vannamei*, *P. stylirostris*, *P. setiferus*, *P. monodon*, and *Metapenaeus ensis* [69,90,101,108]. Natural infections have been reported in *P. vannamei*, *P. setiferus*, and *P. stylirostris* [70,101]. Experimental infections were introduced in *Penaeus chinensis* and the American shrimp species *P. aztecus*, *P. duorarum*, and *P. schmitti*, which can act as potential hosts and carriers of the virus [78,101,109]. It appears that *P. aztecus* and *P. duorarum* are resistant to TSV infection since no clinical signs or histopathological lesions were detected upon experimental infection [109]. It was suggested that TSV may be transmitted vertically [79,93]. After the TSV outbreaks in Latin America, the presence of wild *P. vannamei* stocks with varying levels of TSV resistance was reported. These stocks were used as brooders to stock TSV-resistant postlarvae for shrimp farms in the region. At present, all genetic lines of *P. vannamei* cultured in the Americas and Asia have some degree of resistance to TSV [100].

### 5.4. White Spot Syndrome Virus (WSSV) (Whispovirus Nimaviridae)

This virus first appeared in 1992 in northern Taiwan shrimp farms culturing *P. japonicus*, causing disease and high mortality [110,111]. Soon after, similar diseases appeared throughout Asian countries including China (1993), Japan (1993–94), Korea (1994), Thailand (1994), India (1994), Indonesia (1996), Malaysia (1996), Vietnam (1997), Philippines (1997), and Iran (2002) [112]. Initially it was thought that different viral agents simultaneously appeared in different countries, each giving a specific name to the disease. Later, it was recognized that a single viral agent was responsible for all the outbreaks; hence, an eventual consensus called the pathogen white spot syndrome virus (WSSV) [64,112].

The likely WSSV transmission pathway between Asian countries entailed the movement of live broodstock, nauplii, and postlarvae [113]. On the American continent, WSSV was first recorded in 1995 from *P. setiferus* hatchery facilities in Texas and South Carolina in the U.S. [69,114]. The likely route by which WSSV reached the U.S. was the importation and/or processing of WSSV-infected frozen commodity shrimp from Asian countries [113,115,116].

In 1998, WSSV was reported in Peru, and in 1999, it rapidly spread to many Latin American countries such as Honduras, Ecuador [11,117], Colombia, Panama, Nicaragua, Guatemala, Belize, and Mexico [59,118,119].

In Mexico, WSSV possibly entered through WSSV-infected imported frozen commodity shrimp from Asia [113], since at that time, no importation restrictions existed for frozen commodity shrimp from countries with declared WSSV epizootics [111]. It is also possible that the movement of WSSV-infected live brooders or postlarvae from central America was the source of WSSV [115]. WSSV was first reported in northwest Mexico in 1999 in hatcheries and shrimp farms in Sinaloa [111,120]. In 2001, WSSV-positive shrimp were detected in the coastal areas off northern Sinaloa. It is possible that these WSSV-infected shrimp escaped from nearby farms as a consequence of Hurricane Juliette, which hit this area [120].

**Figure 5:**
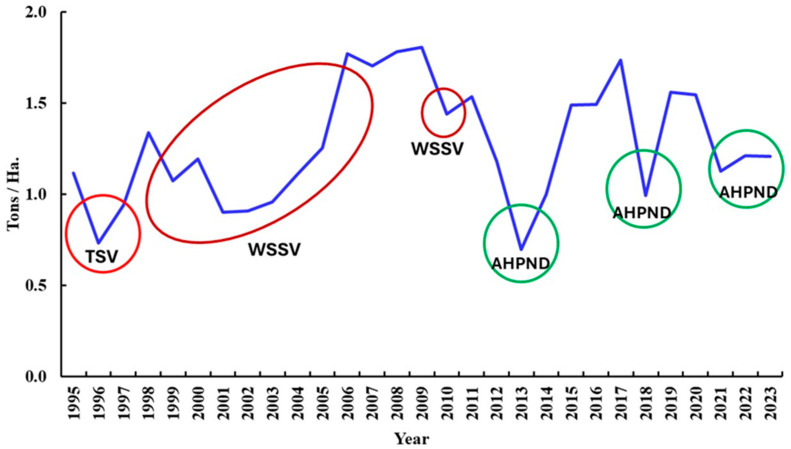
Impact of viral and bacterial diseases in farmed shrimp production in Mexico in the period 1995–2023. Data represent the ratio tons per hectare [6,121]. Here, the impact of diseases can be observed despite the yearly production growth. TSV appeared in Mexico in 1995, but its highest impact occurred in 1996. WSSV appeared in Mexico in 1999 and has been the most damaging pathogen in Mexico causing significant production losses between 2001 and 2005 and 2009 and 2011 (the impact of these viral diseases are circled in red). The toxic disease AHPND caused by *Vibrio parahaemolyticus* appeared in 2013, triggering production losses of 50% that year, and it still caused important losses in 2018 and 2021–2023 (indicated in green circles).

WSSV has become the most damaging virus for farmed shrimp in Mexico. Massive mortality and huge production losses were reported between 2001 and 2005 and again between 2009 and 2011 (Figure 5). Afterwards, another pathogen hit the Mexican shrimp farming industry, replacing WSSV and reducing its impact after 2013.

WSSV has become the most destructive pathogen in shrimp farming worldwide. In 2012, the economic losses estimated since WSSV first appeared in 1992 were USD 6 billion for Asian farming and USD 2 billion for American farming, but it is possible that the actual losses be up to USD 15 billion [113].

WSSV has an enveloped, non-occluded, and bacilliform virion with a size between 210 and 380 nm in length and 70 and 167 nm in width. It has a tail-like appendage at one end of the virion [122,123]. It is one of the largest viruses infecting animals [110,124,125].

The WSSV genome is also one of the largest recorded for viruses infecting animals. It ranges from 293 to 307 kilobase pairs (kbp) [126,127,128]. The genome has between 531 and 683 ORFs encoding peptides from 51 to 6077 amino acids, representing 92% of the genetic information contained in the genome [126,127]. Sequence analysis of the *DNA polymerase* gene and the organization of several ORFs known to encode WSSV structural proteins were different from those of known baculoviruses, demonstrating that WSSV is not closely related to this virus family [126,127,128,129,130]. Thus, WSSV is a new virus, assigned to its own virus family *Nimaviridae* and genus *Whispovirus* [131]. At present, two new viruses infecting wild and farmed crabs in the Sea of Japan (*Chionoecetes opilio* bacilliform virus) (CoBV) [132] and the Pacific coast of Kamchatka (*Chionoecetes bairdi* bacilliform virus) (CbBV) [133] have been determined to belong to the family *Nimaviridae*. These two viruses may be variants of a new species of *Nimaviridae*. Another study found ancestral genomes of *Nimaviridae* inserted within genomes of crabs, indicating that members of this family have been infecting crustaceans for a long time [134].

**Figure 6:**
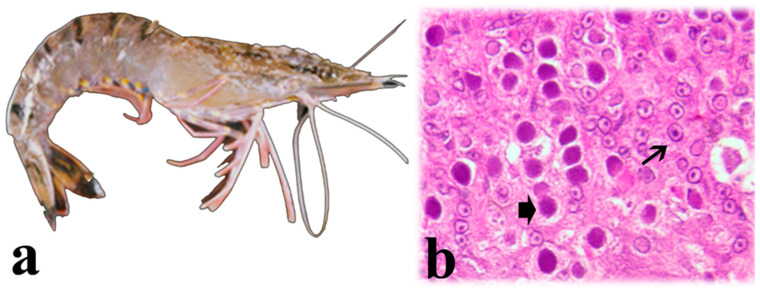
WSSV infection in shrimp. (**a**) Black tiger prawn (*P. monodon*) displaying white spots on the surface of the exoskeleton at the head and tail. In Pacific white shrimp (*P. vannamei*), obvious white spots on the exoskeleton are seldom observed. (**b**) Histopathology of WSSV infection in connective tissues of the stomach. Cowdry A-type inclusion bodies indicating early WSSV infection in cells (narrow arrow). Nuclear hypertrophy with basophilic inclusion bodies in cells (wide arrow) indicating advanced WSSV infection in cells. Tissue displays areas of vacuolized necrosis (void spaces around cells). Magnification 400×. Own images.

Clinical signs of WSSV infection in Asian shrimp species are the presence of white spots in the inner surface of the exoskeleton (Figure 6a) [119,135,136], whereas white spots are not easily visible in WSSV-infected *P. vannamei*. These are probably the accumulation of calcium salts resulting from the vacuolization of the cuticular epithelium [137]. Other clinical signs include reddish discoloration of the body and appendages [130,138], reduced feeding, preening, and response to stimuli [110,122,123,139,140], a loose cuticle [135], and the enlargement and discoloration of the hepatopancreas [141]. Around one or two months after pond stocking [136], disease becomes apparent and WSSV-infected shrimp gather near the pond edge one or two days before mortality occurs [142]. Cumulative mortality may reach up to 100% within 10 days after the onset of clinical signs of disease [143,144].

Histopathology analyses show WSSV-infected cells with hypertrophied nuclei, amphophilic intranuclear inclusions, and marginated chromatin [139,145]. Intranuclear inclusions are markedly distinct and bigger than the cowdry A-type inclusions displayed in *Penstylhamaparvovirus* (IHHNV) infection [122,136]. Infected nuclei progressively increase in size and become basophilic [123,125,135,139,140,146]. In the late stages of infection, karyorrhexis and cellular disintegration may occur, leading to the formation of necrotic vacuolized areas [136,137] (Figure 6b).

Susceptible species to natural or experimental infections include penaeid shrimp (*P. aztecus*, *P. duorarum*, *P. chinensis*, *P. indicus*, *P. merguiensis*, *P. setiferus*, *P. stylirostris*, *P. vannamei*, *P japonicus*, *P. monodon*, *P. penicillatus*, *P. semisulcatus*, *Metapenaeus dobsonii*, *M. ensis*, *M. monoceros*, *Parapenaeopsis stylifera*, *Solenocera indica*, and *Trachypenaeus curvirostris*), caridean shrimp, lobsters, crayfish, crabs, and other decapod crustaceans [112]. Shrimp postlarvae, juveniles [147], and adults [148,149] are susceptible to WSSV infection.

### 5.5. Yellow-Head Virus–Gill Associated-Virus (YHV-GAV Complex: Roniviridae)

This pathogen has up to six different genotypes, of which only genotype 1 (*Okavirus* 1), Yellowhead Virus and Gill-Associated Virus (GAV) from Australia, is associated with disease [13,150,151,152]. YHV first appeared in 1990 in shrimp farms culturing *P. monodon* in Thailand [153]. Its name describes the signs of the disease, including the light-yellow discoloration of the cephalothorax due to the swelling and color change in the hepatopancreas and gills. In American species, yellow discoloration of the head does not occur [13]; instead, a generalized pale discoloration of the body occurs [80].

It is possible that YHV caused disease and mortality to farmed shrimp in Taiwan as early as 1986–1987 [64]. Soon after YHV appeared in Thailand, it spread to other Asian countries including China (1989), Philippines (1989), India (1994), Indonesia (1994), Malaysia (1994), Vietnam, Sri Lanka (1998), and Madagascar [78,154,155,156]. The YHV strain known as GAV was reported in farmed *P. monodon* displaying disease and mortality in Australia in 1994 [157].

The virus reached America, entering the U.S. in 1995. It is possible that YHV was introduced in the American continent by frozen commodity shrimp products, just like WSSV [43,78,80,113,115]. In Mexico, YHV was detected in 1999 in some shrimp farms located in the western states of Sonora, Sinaloa, and Nayarit [150,155,158]. It is possible that both WSSV and YHV entered Mexico at around the same time either through infected frozen commodity shrimp or through infected broodstock or postlarvae into hatcheries or grow-out farms, but WSSV was far more pathogenic to *P. vannamei* and *P. stylirostris* than YHV, overshadowing it. In 2003, the presence of YHV was determined by RT-PCR at low prevalence (0.02%) in wild populations of *P. stylirostris* off the coast of Guasave, Sinaloa [159]. In 2009, YHV was also detected at low prevalence (13.3%) and with low pathogenicity in two freshwater shrimp farms culturing *P. vannamei* at Colima, Mexico. The only effect of YHV infection reported in farmed shrimp was stunted growth since shrimp stopped growing at 4 g after 65 days cultured. No patent histopathological signs of YHV were observed in the lymphoid organ, and only few cells with pyknosis and karyorrhexis were observed in gill filaments [160]. It is possible that the Mexican YHV isolates had low pathogenicity since they showed low prevalence and did not cause tissue damage as extensive as that reported in Asian shrimp species.

Morphologically, the YHV virion is enveloped and bacilliform with a size of 150–200 nm long and 40–50 nm wide [161,162]. Due to its shape, it was first thought to belong to the granulosis-like virus (*Baculoviridae*). Later, its genomic organization was determined to be a single-stranded (positive-strand) RNA molecule [163,164]. The complete genome is a linear single-stranded RNA molecule of 26,652 nucleotides [164]. The genome is organized into four distinct ORFs. ORF1a has a 3C-like protease motif, whereas ORF1b has a “SDD” polymerase metal ion-binding domain helicase. ORF2 encodes putative nucleocapsid proteins (g7 and g2) and ORF3 encodes putative surface glycoproteins (p18/20, p33 and g2.1 [152,164]).

The taxonomic position of the YHV-GAV complex has been changing. At first, YHV was thought to be a baculovirus due to its size and shape and the presence of a membranous envelope [150,153]. Later, it was found that its genome was a single-stranded RNA molecule, placing it either as a rhabdovirus or a coronavirus [163]. Then, its RNA strand was reported to have negative polarity, placing it in the family *Rhabdoviridae* [162], but later, it was reported as a positive-polarity RNA strand by *in situ* hybridization and sequencing, hence being classified as a corona-like virus [150,163,165]. Later, it was found that YHV produced a dsRNA replicative intermediate and the polyproteins encoded by ORF1a and ORF1ab were translated by an N1 frameshift [166]. This trait indicated that YHV-GAV belongs to the Order Nidovirales as its first member infecting invertebrates. As YHV-GAV primarily infects the lymphoid (Oka) organ, the new proposed genus name is *Okavirus* within the new family *Roniviridae* [152,166]. Members of the *Roniviridae* are closely related to other Nidoviruses infecting mosquitoes (*Mesoniviridae*) and crustaceans (*Euroniviridae*) [152].

**Figure 7:**
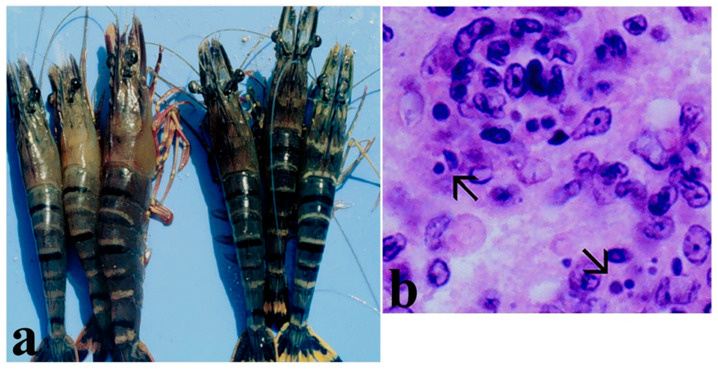
Clinical signs and histopathology of YHV infection. (**a**) Black tiger prawn *P. monodon* with Yellow Head Disease (YHD) (left). Shrimp display yellowish discoloration of the head and appendages. On the right, non-infected shrimp with normal body color. (**b**) Histopathology of the lymphoid organ (LO) of a juvenile giant black tiger shrimp with acute YHD. Affected cells of LO display pyknotic and karyorrhectic nuclei and LO shows extensive diffuse necrosis. Infected cells present single or multiple perinuclear pale-to-dark basophilic inclusion bodies (arrows). YHV differential diagnosis from TSV infection is the presence of marked necrosis in LO during acute infection. TSV produces similar cytopathology in other organs except LO. Magnification 1700× (photos from AGDAF-NACA 2007) [60].

Clinical signs of YHV infection include pale-yellow body discoloration, especially yellowish hepatopancreas and gills in *P. monodon* (Figure 7a). Other signs include erratic swimming near pond shores and cumulative mortality up to 100% within 3–5 days after the onset of clinical signs [80,155,161]. YHV causes systemic infection and replicates in tissues and organs of ectodermic and mesodermic origin such as the gills, digestive tract, lymphoid organ, hematopoietic tissues, connective tissues of subcutis, antennal gland, gonads, nerves, ganglia, eyestalk, hepatopancreas, and muscle [155,167]. Histopathology shows basophilic, perinuclear cytoplasmic inclusion bodies, along with pyknosis and kariorrhexis in cells of epithelia and connective tissues of affected organs (Figure 7b) [138,156].

YHV affects early to late juvenile (1–15 g) shrimp stages. In the pond, infected shrimp display abnormally fast feed consumption for several days then suddenly stop feeding, and the first moribund shrimp appear swimming slowly near the surface at the edge of ponds. The next day the number of moribund shrimp increases, and by the third day after the onset of disease, 80–100% of shrimp die. Dead shrimp are evenly scattered at the edge and the bottom of the ponds [153,156].

Natural infections and experimental challenge assays have shown that YHV-susceptible species include *P. monodon*, *P. merguiensis*, *M. ensis*, *P. vannamei*, *P. stylirostris*, *P. setiferus*, *P. duorarum*, and *P. aztecus*, as well as the freshwater prawns *Palaemon styliferus*, *Palaemonetes pugio*, and *Acetes* sp. [69,138,168]. The estimated losses caused by YHV from 1990 to 2007 are USD 500 million [168].

### 5.6. Necrotizing Hepatopancreatitis (NHP) (Hepatobacter penaei)

This type of bacteria belongs to the Order Rickettsiales. They are prokaryotic intracellular pleomorphic pathogens with a Gram-negative cell wall [53]. These pathogens reproduce by binary fission, and some members have been described to infect mollusks and crustaceans, including marine crabs and many species of penaeid shrimp [53].

The first rickettsia bacteria infecting crustaceans were reported in 1970 in a terrestrial isopod [169]. Rickettsial infections in marine crustaceans were reported in connective tissues of the marine crab *Carcinus mediterraneus* in 1980 [53,170]. In 1984, rickettsial infections were reported in the hepatopancreas epithelium of the crab *Paralithodes platypus* and in the hepatopancreas of farmed shrimp *P. merguiensis* from Singapore and cage-cultured in Malaysia [170]. In 1986, the hepatopancreas of wild shrimp *P. marginatus* from Hawaii showed the presence of rickettsia. In this species rickettsia infection resulted in a mild disease with no external clinical signs. In 1987, a systemic rickettsial infection was reported in farmed *P. monodon* from Malaysia. Heavy bacterial infections were widespread in ectodermal and mesodermal tissues but did not spread to endodermal tissues. The systemic rickettsial infection was limited to *P. monodon* from Malaysia, and its experimental transmission was not reported to other shrimp species. The systemic nature of this rickettsia infection contrasts with the rickettsial infection confined to epithelial cells of hepatopancreas reported in most penaeid shrimp species [53,170].

In the American continent, necrotizing hepatopancreatitis (NHP) was first reported in farmed *P. vannamei* and *P. aztecus* from Texas in 1985 [171,172,173,174]. Later, the disease was found in farmed *P. vannamei* on the Pacific coast of Peru in early 1993, which was the first report of the disease outside the state of Texas [174,175,176]. Later, NHP was reported to cause disease in other Latin American countries, such as Ecuador, Venezuela, Brazil, Panama, Mexico, Belize, Guatemala, Nicaragua, El Salvador, Costa Rica, and Colombia, and in Asia, in Vietnam [69,177,178].

In Mexico, NHP was first reported in 1999 in farmed *P. vannamei* and *P. stylirostris* from Sonora and Sinaloa, where the disease caused mortality between 20% and 80% during the summer when the water temperature was ≥34 °C and salinity reached 45 g/kg [179].

The disease caused by NHP is known as Texas necrotizing hepatopancreatitis (TNHP), the Texas pond mortality syndrome, Peru necrotizing hepatopancreatitis (PNHP), Ecuador necrotizing hepatopancreatitis (ENHP), NHP bacterium (NHPB), rickettsial-like organism (RLO), and granulomatous hepatopancreatitis [175,178,180].

In Texas shrimp farms the disease occurs seasonally and has frequently caused serious yield losses (20 to 90%) [172,175]. In the Americas, NHP disease appears with the combination of high-water temperature (≥−30 °C) and high salinity (≥40 g/kg) for many days [69,113,172,173,181]. Conversely, the major shrimp-producing countries in Asia have not reported the presence of NHP despite the frequent introduction of potentially infected stocks of *P. vannamei*. This suggests that NHP manifestation involves a high water temperature and high salinity for some time during the dry season [113].

NHP is a disease that affects juvenile and adult stages of *P. vannamei*. Damages in epithelial cells of hepatopancreas have been reported in other penaeid species including *P. aztecus*, *P. setiferus*, *P. stylirostris*, and *P. californiensis*. NHP has also been reported in several penaeids from brackish and marine waters [69,181].

**Figure 8:**
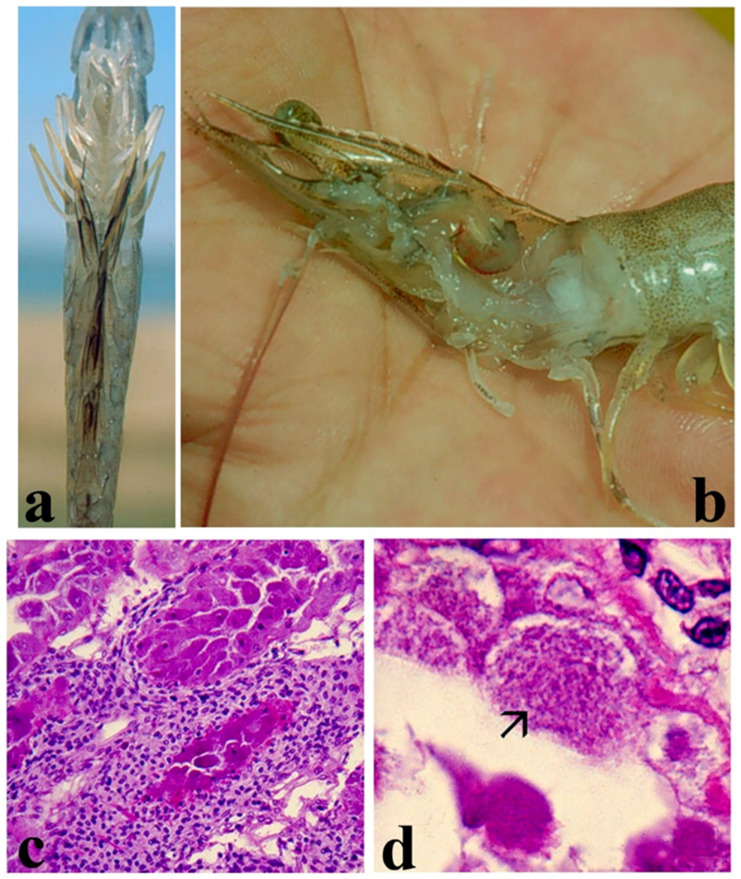
(**a**) Shrimp *P. vannamei* affected by necrotizing hepatopancreatitis (NHP). The edges and base of pleopods appear darkened due to melanization. (**b**) Juvenile *P. vannamei* affected by NHP. Hepatopancreas is reduced in size due to atrophy and appears whitish and soft. (**c**) Histopathological changes in hepatopancreas of juvenile *P. vannamei* with severe, subacute (grade 3–4) necrotizing hepatopancreatitis (NHP). Hemocytic infiltration, cytolysis, and sloughing of HP tubule epithelial cells are the main histological changes due to NHP (magnification 300×). (**d**) Epithelial cells of hepatopancreas showing no cytoplasmic lipid droplets; instead, they are filled with non-membrane-bound intracytoplasmic NHP bacteria (arrow) (magnification 1700×) (all images from AGDAF-NACA 2007) [60].

Clinical signs include lethargy, weakness, atrophy of hepatopancreas, reduced feeding, empty gut, soft exoskeleton, flaccid body, surface body fouling by epicommensal microorganisms, and darkened gills and pleopods [177] (Figure 8). Mortality rates range from 20 to 95%. These signs are nonspecific and do not provide a definitive diagnosis of NHP [175,176].

The NHP target tissue is the hepatopancreas, affecting all hepatopancreatic cell types. In moribund shrimp the hepatopancreas becomes pale white, with atrophied hepatopancreatic tubules, marked lipid reduction due to a decreased number or the absence of resorptive (R) cells and blister-like (B) cells, and multifocal granulomas [170,172,175,176,177] (Figure 8). In granulomatous tissues, cells may be hypertrophied, showing abundant free, pale, basophilic, NHP bacteria in the cytoplasm. The nuclei of hypertrophied cells may be normal or pycnotic. In severe NHP infection the pale tubules of hepatopancreas become melanized, displaying black streaks, and the hepatopancreas turns soft and fills with fluid [69,177]. Secondary focal or systemic *Vibrio* infections have been observed in severe NHP infection [177].

The NHP bacterium has a complex life cycle with at least two morphological development stages within infected hepatopancreatic tubular epithelial cells [172,175,176]. The NHP agent is a Gram-negative, intracellular rickettsia-like bacterium living free within the cytoplasm of infected hepatopancreatic cells. The predominant form is small, pleomorphic, rod-shaped, and rickettsia-like (0.25–0.9 µm), and there is a less common helical form (0.25–2–3.5 µm) with eight flagella at the basal apex [170,172,175,176,180].

Three major developmental stages of NHP disease have been characterized [172]:

Stage I—The presence of small, pleomorphic, intracytoplasmic individual bacteria or patches of bacteria is observed in adjacent tubular epithelial cells within a tubule. Bacteria are limited to the tubular epithelium and are present in the apical cytoplasm or fill the cytoplasm of individual hypertrophic cells. Hemocytic infiltration of granular and hyaline hemocytes is absent except when necrotic tubules appear.

Stage II—The hypertrophy of epithelial cells of tubules leads to tubular dilatation and obliteration of the lumen. These cells are filled with numerous Gram-negative bacteria and often show cytoplasmic protrusions filled with bacteria that extend to the lumina. At this stage intertubular sinus dilatation and hemocytic congestion are present, but limited distal tubular necrosis is observed. Desquamation and necrosis occur, and individual epithelial cells slough into the tubule lumen. The lipid content of epithelial cells decreases, and massive clumps of bacteria are observed at the center of necrotic tubules.

Stage III—Areas of necrotic distal tubules increase and extend to medial and proximal segments. Huge numbers of intracellular bacteria are present in tubular epithelial cells. Necrotic tubules collapse, containing intraluminal hemocytes, and display a thickened, fragmented basal membrane. Lamellar peritubular interstitial fibrosis and melanization are observed, and tubules show moderate to severe dilation and epithelial reduction.

Experimental infection with NHP in *P. stylisrostris* produced infection limited to epithelial cells of the hepatopancreas but triggered an acute potentially lethal disease [53,170]. In experimental infections in *P. vannamei*, development of stage I disease was reported at 6–23 days post-exposure, stage II at 16–37 days post-exposure, and stage III at 16–51 days post-exposure. Stage III was the most destructive, and its appearance corresponded to the highest mortality observed [177].

Sequence analysis of the16S rRNA gene of NHP revealed that the agent is an unclassified alpha-Proteobacterium [177,180]. Phylogenetic analyses indicated that it is closely related to bacterial endosymbionts of protozoa, *Caedibacter caryophila* and *Holospora obtusa* [180]. Further sequencing and phylogenetic analyses of the 16S rRNA and gyrase B genes showed that NHP is a member of the order Rickettsiales and its proposed scientific name is *Hepatobacterium penaei* [113,177,182].

### 5.7. AHPND (Acute Hepatopancreas Necrosis Disease) (Vibrio parahaemolyticus and Other Species)

Bacteria are ubiquitous microorganisms in aquatic environments. When faced with environmental changes and a lack of nutriments, some free-living bacteria can modify their habits by becoming opportunistic pathogens, causing disease in animals with a weakened defense system [183]. Bacteria of the family Vibrionaceae are among the most common aquatic bacteria. These can be found in marine and brackish-water environments, so they can tolerate a broad salinity range, with the optimum salinity being 2.0–2.5%. Vibrios are heterotrophic, mesophile, facultative anaerobic, Gamma-proteobacteria, Gram-negative, oxidase-positive microorganisms, and they are generally motile with the presence of a single polar flagellum [184].

Members of the genus *Vibrio* have often been reported as opportunistic, including *V. parahaemolyticus*, *V. harveyi*, *V. vulnificus*, *V. alginolyticus*, and many others, since they develop pathogenic variants in aquaculture systems [185]. Environmental variations in dissolved oxygen, temperature, salinity, pH, organic matter, or the presence of pollutants often trigger stress in many farmed aquatic animals, provoking physiological imbalances and weakening their defense system. This prompts *Vibrio* species to colonize stressed animals, becoming pathogenic causing disease and mortality [183,184,185]. The species *V. parahaemolyticus* and *V. vulnificus* can also be zoonotic pathogens, associated with human food poisoning and septicemia through the ingestion of contaminated seafood [186].

Isolates of *Vibrio parahaemolyticus* have been related to a novel disease affecting farmed shrimp. A next-generation sequencing platform was used to determine the presence of a 69–70 kilobase pair (kbp) extrachromosomal plasmid (PVA-1) in all the AHPND-positive *V. parahaemolyticus* analyzed but not in the AHPND-negative isolates. Researchers also found the presence of an operon composed of ORFs 50 and 51, which encoded a homolog of the *Photorhabdus* insect-related (Pir) binary toxin PirAB [187]. This homology suggested that the binary toxin PirAB may exhibit pore-forming activity. It was also found that the structure of PirAB is analog to the Cry toxin of *Bacillus thuringiensis*, another pore-forming toxin in insects [187,188]. These toxins induce cell death through a post-segregation system, and it spreads by conjugative transfer [187,189]. This disease was first reported in 2009 in China and rapidly spread to other countries in southeast Asia including Vietnam (2010), Malaysia (2011), Thailand (2012), Bangladesh (2013), and the Philippines (2014) [189,190,191,192,193]. AHPND has caused huge economic losses worldwide. In Asian countries the yearly losses were estimated at USD 1 billion [194], but in 2021, the estimated economic impact of AHPND in Asian countries (China, Malaysia, Thailand, Vietnam) and Mexico was USD 43 billion [195].

In 2013, many shrimp farms in northwest Mexico experienced mortalities that did not correspond with WSSV, which was the main pathogen at the time. These mortalities were first observed in Nayarit State (south of the region). Here, water temperatures rise early in the year (February), and it is the first area to begin the shrimp farming season. In early March, the states of Sinaloa and Sonora began stocking ponds with postlarvae, and soon after, in May and June the first massive mortalities were reported, decreasing in the following months [196,197]. Most mortalities reported in this season were not related to WSSV since PCR testing gave negative results, indicating the presence of a novel unknown agent. In both states, some farms had cumulative mortalities up to 95% [196,197]. In 2014, AHPND spread further south to Chiapas state, where it caused up to 95% production losses. The likely transmission pathway was the movement of AHPND-contaminated live postlarvae from a hatchery in Sinaloa [198]. The presence of AHPND gradually increased in shrimp farms in Mexico, becoming the main lethal disease by 2015, downgrading WSSV.

In 2013, the total farmed shrimp production in Mexico was 60% (60,191 tons) of that obtained the previous year (100,321 tons) (Figure 1). The economic losses caused by AHPND were estimated at USD 41 million in 2013 alone [199]. The main hypothesis for the introduction of AHPND into Mexico is through the movement of live shrimp from an AHPND-affected Asian country [200]. Since then, AHPND has been reported in South America (2016) [192,201] and Costa Rica, in 2017 [202].

From its first appearance in China until 2011, the etiology was unknown, and experimental work did not find an infectious agent, so the disease was called “Early Mortality Syndrome” (EMS). In 2011, bioassays inoculating cell-free supernatants of AHPND cultures in shrimp by reverse gavage displayed EMS clinical signs and displayed a unique histopathology, indicating the presence of a toxin. Histopathology showed massive sloughing of epithelial cells in hepatopancreas, from the central part of the tubule outwards to the distal part. This desquamation happened in the absence of any observed pathogen. Due to this feature during the acute phase of the disease, it was called “acute hepatopancreatic necrosis syndrome” (AHPNS) [46,189,190]. In 2013, the etiological agent was isolated in pure culture, and it was identified as a *Vibrio parahaemolyticus* carrying a plasmid containing genes encoding a binary toxin; thus, the disease was called “acute hepatopancreatic necrosis disease” (AHPND) [187,190].

**Figure 9:**
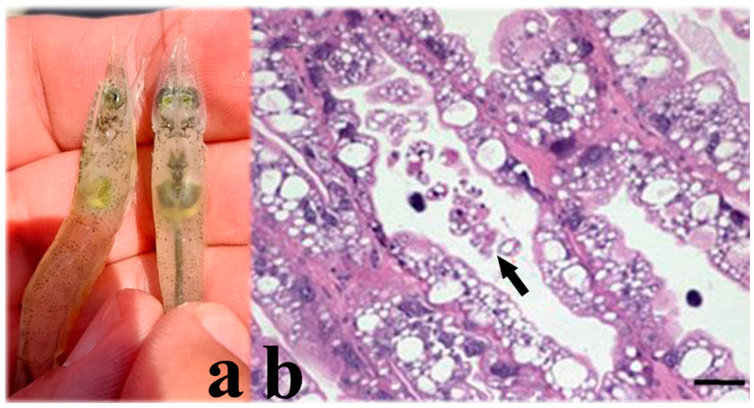
(**a**) Clinical signs of AHPND in farmed *P. vannamei*. The shrimp on the left is affected by AHPND and displays an atrophied pale hepatopancreas and empty gut. The shrimp on the right is unaffected and shows a normal-sized hepatopancreas without discoloration and a full gut (own photo). (**b**) Histopathology of the hepatopancreas of shrimp affected by AHPND in the acute phase. Sloughing of tubular epithelial cells (arrow) into the tubule lumen is observed (scale bar = 25 μm) (magnification 400×) (photograph taken from Tang et al. 2020) [215].

AHPND appears in farmed shrimp between 20 and 35 days post stocking. The disease has been reported as early as 7–10 days after stocking. Postlarvae and early juveniles (≤3 g) are the life stages most affected by AHPND [188], but sometimes juvenile shrimp ~10 g have also been affected. Infection starts when bacteria enter the digestive tract through ingestion and colonize the hepatopancreas and midgut. Here, they secrete the toxins, causing massive desquamation and sloughing of epithelial cells of the hepatopancreas and necrosis of tubules, inducing hemocytic infiltration (Figure 9) [46,198,203]. Clinical signs of disease include atrophy and pale discoloration of the hepatopancreas, often displaying dark streaks, a lack of feeding, an empty gut or discontinuous gut content (Figure 9), a soft exoskeleton, reduced activity, ataxia and erratic swimming, the presence of secondary bacterial infections, and massive death up to 60 days after stocking [193,196,204].

Three stages of AHPND infection are recognized based on histopathology: initial, acute, and terminal [195,197]. In the initial stage, epithelial cells become enlarged and protrude into the lumen of the tubule, reducing the size of the vacuoles of R and B cells, and epithelial cells slough into the tubule lumen. The acute stage displays desquamation of the tubular epithelium, and dead cells accumulate in the lumen. Necrosis of the hepatopancreas spreads from the proximal end to the distal end of tubules, with hemocytic infiltration in response to necrosis. In the terminal stage, intertubular connective tissues display strong inflammation, with hemocytic infiltration in the interstitial spaces of tubules and encapsulation around tubules. Advanced necrosis and melanization are observed in tubules, and massive bacterial proliferation occurs inside the tubular lumina.

The environmental factors that may trigger AHPND in endemic areas include high water temperature (≥28 °C) during the dry season from April to July, salinity ≥ 29 g/kg, dissolved oxygen ~5 ppm, and pH > 7. Other factors include low water exchange along with low planktonic biodiversity, a high concentration of soluble nutrients in water by the addition of fertilizers or molasses, and the accumulation of organic matter in sediments as a result of overfeeding, algal crashes, etc. [100,205,206].

In 2009, the species *V. parahaemolyticus* was the only one associated with AHPND, but since then, other vibrios have been reported to harbor the plasmid containing the PirA/B toxin genes. These are *V. harveyi* [201,207], *V. campbelli* [208,209], *V. owensi* [210], *V. punensis* [192], and *V. shilonii* [211]. The species *V. parahaemolyticus*, *V. harveyi*, *V. campbelli*, and *V. owensii* belong to the Vibrio clade, but the range of transmission of the AHPND toxic genes has reached other species such as *V. shilonii* (=*V. mediterranei*), which belongs to the Mediterranei clade, members of which are either pathogenic to corals, causing coral bleaching in the Atlantic ocean and Mediterranean sea, or pathogenic to shell clams [212,213]. Moreover, the species *V. punensis* is a member of the clade Orientalis, which contains non-pathogenic *Vibrio* species that are probiotic in natural conditions [188,192]. These findings stress the concern that the AHPND genes may spread to other pathogenic and non-pathogenic clades of *Vibrio* species, threatening other marine species and ecological communities.

The shrimp species that are susceptible to AHPND include *P. vannamei*, *P. monodon*, the fleshy prawn *P. chinensis* [214], and the palaemonid freshwater prawn *Macrobrachium rosenbergii* [195].

Mexico is one of the main shrimp farming producers in America and worldwide. Trading living shrimp either as brooders or larval stages for farming purposes or importing frozen shrimp commodities for human consumption has the risk of carrying infectious pathogens or diseases to new areas [11,113,117,200,216]. It is likely that any of these actions allowed entry to some of the pathogens that have impacted the Mexican shrimp farming industry. The enforcement of border biosecurity measures such as quarantine protocols and the application of highly sensitive, specific diagnostic techniques are required to reduce the risk of entry to other emerging pathogens such as *Enterocytozoon hepatopenaei* (EHP) [217], white feces disease [218], Decapod Iridescent Virus 1 (DIV1) [219], or Covert Mortality Nodavirus [220]. Countries such as Brazil and Venezuela have applied strict border controls to frozen crustacean commodities for human consumption [11,22] and have developed domestication programs to prevent the entry of exotic pathogens through live animals [11]. Likewise, Mexico also strengthened its border controls to prevent the entry of live and frozen crustacean commodities after AHPND appeared in 2013 [221].

The way in which diseases have occurred in Mexico may be somewhat different to other parts of the world. The major pathogens have become dominant during a specific time period, briefly coexisting with a new pathogen as it becomes established, until the former pathogen is gradually replaced by the new one, which becomes the main disease agent. Except for *Penstylhamaparvovirus* (IHHNV), which has persisted both in shrimp farming operations and wild populations apparently without causing major disease or mortality, most of the pathogens that have arrived in Mexico have not coexisted in Mexican shrimp farms.

Domesticated specific-pathogen-resistant (SPR) stocks of penaeid shrimp have been used in Mexico since the TSV outbreak in 1996 [11,79]. Efforts to develop genetic improvement programs to produce new shrimp lines resistant to pathogens such as WSSV have been made by public institutions and private companies worldwide. In Mexico, a private company developed *P. vannamei* shrimp lines with improved resistance to WSSV [222], as well as evaluating the heritability of AHPND resistance in shrimp lines in Mexico [223]. It is envisioned that similar programs will arise worldwide to generate transgenic shrimp lines with multiple resistance to viral and bacterial pathogens [224]. Sustainable options for disease control and improved survival rate, defense response, and enhanced growth may include the use of bacterial-derived additives. Evidence shows that animal gut microbiota can be modulated by functional additives in the form of probiotics, synbiotics, paraprobiotics, and postbiotics to thwart bacterial diseases such as AHPND, at the same time as contributing to reducing the use of antibiotics [225]. Shrimp production in Mexico will have to become more technologically efficient and sustainable to deal with infectious diseases to increase production.

## Figures and Tables

**Table 1:** Main shrimp farming countries in 2023 [4].

Ranking	Country	Volume (Tons)	Species	Percentage
1	China	2,575,789	*P. vannamei* *P. monodon* *P. japonicus*	31.6
2	India	1,240,646	*P. vannamei* *P. monodon*	15.3
3	Ecuador	1,220,200	*P. vannamei*	15.0
4	Vietnam	1,167,383	*P. vannamei* *P. monodon*	14.4
5	Indonesia	934,825	*P. vannamei* *P. monodon* *P. merguiensis*	11.5
6	Thailand	392,470	*P. vannamei* *P. monodon* *P. merguiensis*	4.8
7	Mexico	194,066	*P. vannamei* *P. stylirostris*	2.4
8	Brazil	127,466	*P. vannamei*	1.6
9	Bangladesh	86,079	*P. monodon*	1.0
10	Philippines	64,273	*P. vannamei* *P. monodon* *P. merguiensis*	0.8
11	Malaysia	54,379	*P. vannamei* *P. monodon*	0.7
12	Peru	42,927	*P. vannamei*	0.5
13	Nicaragua	32,000	*P. vannamei*	0.4
	Total	8,132,503		100.0

## Data Availability

No new data were created or analyzed in this study. Data sharing is not applicable to this article.
